# *dmrt1* Is Responsible for Androgen-Induced Masculinization in Nile Tilapia

**DOI:** 10.3390/genes15091238

**Published:** 2024-09-23

**Authors:** Shengfei Dai, Mei Li, Jie Yuan, Xueyan Wei, Eryan Ma, Deshou Wang, Minghui Li

**Affiliations:** Integrative Science Center of Germplasm Creation in Western China (CHONGQING) Science City, Key Laboratory of Freshwater Fish Reproduction and Development (Ministry of Education), Key Laboratory of Chongqing Municipality for Aquatic Economic Animal Resources Conservation and Germplasm Creation, School of Life Sciences, Southwest University, Chongqing 400715, China; 15123991935@163.com (S.D.); 15095874760@163.com (M.L.); 18090539785@163.com (J.Y.); 13350384509@163.com (X.W.); 15035804079@163.com (E.M.); wdeshou@swu.edu.cn (D.W.)

**Keywords:** Nile tilapia, 17α-methyltestosterone, masculinization, sex reversal, *dmrt1*

## Abstract

17α-Methyltestosterone (MT) is a widely used androgen for all-male fish production in aquaculture. However, the molecular mechanism underlying MT-induced masculinization remains unclear. In this study, we aim to identify the key gene responsible for MT-induced masculinization using the Nile tilapia (*Oreochromis niloticus*) *amhy*, *dmrt1*, and *gsdf* mutants, which exhibit male-to-female sex reversal. Nile tilapia fry from these three mutant lines were treated with 50 μg/g MT from 5 to 30 days after hatching (dah). The results showed that *amhy* and *gsdf* mutants, but not *dmrt1* mutants, were masculinized by the MT treatment. Gonadal transcriptome analysis revealed that genes involved in steroidogenesis and germ cell development in MT-treated *dmrt1* mutants exhibited a similar expression pattern to that of the wild type (WT) XX. In addition, the *dmrt1* mutants cannot be masculinized by co-treatment with MT and the aromatase inhibitor fadrozole. The MT treatment completely blocked early steroidogenic enzyme (Star2, Cyp17a2, and Cyp19a1a) expression independent of *amhy*, *gsdf*, and *dmrt1*. A luciferase analysis showed that MT directly suppressed basal and Sf-1-activated *cyp19a1a* promoter activity through *ara* and *arb* in cultured HEK293 cells. Furthermore, MT treatment inhibited germ cell proliferation in *amhy* and *gsdf* mutants but not in *dmrt1* mutants. Consistently, *dmrt1* expression was induced in MT-treated WT XX, -*amhy*, and -*gsdf* mutants. Taken together, these results suggest that *dmrt1* is indispensable for MT-induced masculinization in Nile tilapia and that MT functions by inhibiting early steroid synthesis and activating *dmrt1* to promote testis development.

## 1. Introduction

Sex determination (SD) is the process by which a bipotential gonad is directed to develop into either an ovary or a testis [1]. In teleosts, SD is triggered by a variable master sex-determining gene, and then sex differentiation is regulated by a more or less conserved downstream regulatory network [2]. Even with a stable SD, the teleost gonad displays a high degree of sexual plasticity. Environmental factors other than genetic cues, such as temperature, nutrients, social status, body size, and exogenous sex hormones, can influence fish sexual differentiation [3]. Among the hormones used to induce masculinization in genetic female fish, 17α-methyltestosterone (MT) is the most widely used androgen. MT has been successfully employed to induce masculinization in a number of fish, including Japanese medaka (*Oryzias latipes*) [4], Nile tilapia (*Oreochromis niloticus*) [5], common carp (*Cyprinus carpio* L.) [6], rainbow trout (*Oncorhynchus mykiss*) [7], zebrafish (*Danio rerio*) [8], mangrove killifish (*Kryptolebias marmoratus*) [9], yellow drum (*Nibea albiflora*) [10], mandarin fish (*Siniperca chuatsi*) [11], etc. However, the molecular mechanism of MT-induced masculinization remains unclear so far.

It is well known that endogenous estrogens, which are converted from androgen by aromatase encoding by *cyp19a1a/b*, are natural inducers for ovarian differentiation and maintenance in fish [3]. Treatment with aromatase inhibitor fadrozole/letrozole or mutation of *cyp11a2*, *cyp17a1*, and *cyp19a1a* resulted in female-to-male sex reversal in fish [12,13,14]. During MT treatment, the expression of steroidogenic enzymes (Cyp11a1, 3β-HSD, Cyp19a1a) responsible for estrogen production and the female pathway gene *foxl2* were inhibited, and conversely, male pathway genes, such as *amh*, *gsdf*, and *dmrt1*, were up-regulated, as reported in mandarin fish [11], Nile tilapia [15,16], northern medaka (*Oryzias sakaizumii*) [17], and zebrafish [18]. It is well known that male and female pathway genes play antagonistic roles in vertebrate sexual differentiation. For example, Dmrt1 directly binds to the *cyp19a1a* promoter and inhibits its transcription in male Nile tilapia [19]. In fact, the *dmrt1* and *cyp19a1a* genes showed opposite expression patterns in male and female Nile tilapia gonads, with *dmrt1* high in testes and *cyp19a1a* high in ovaries. This is also true in the fin tissue and could therefore be used to identify sex in live Nile tilapia [20]. However, it is unknown whether the inhibition of steroidogenic enzyme expression by MT treatment is a direct effect or an indirect effect that depends on the activation of the male pathway genes.

On the other hand, it has been reported that reduced germ cell numbers in XX females upon MT treatment were observed in northern medaka [17] and Japanese medaka [21]. In Nile tilapia, the number of germ cells differs between females and males between 9 and 15 dah. In XX females, germ cells continue to undergo mitosis division from 9 dah, whereas in XY males, germ cells enter a short period of quiescence and resume proliferation by 15 dah [22]. Consistently, this period (9–15 dah) also overlaps with the time when male pathway genes, such as *dmrt1* and *amh*, are up-regulated in XY gonads [23]. Therefore, it is possible that the up-regulated male pathway genes hinder germ cell proliferation in the XY gonads. It is also unclear whether the MT-induced suppression of germ cell proliferation relies on male pathway genes.

The Nile tilapia, *O. niloticus*, is a gonochoristic teleost fish with a male heterogametic (XX/XY) SD system. The male pathway genes of Nile tilapia include *amhy*, *dmrt1*, and *gsdf*. Among them, *amhy* serves as the male sex initiator, while *dmrt1* and *gsdf* function as male sex maintainers (Supplementary Figure S1). In recent years, with the establishment of genome editing technologies, several mutants of male and female pathway genes were established in this species, including the master sex-determining genes *amhy* [24], *gsdf* [25], *dmrt1* [26], *foxl2*, and *cyp19a1a* [27]. These advances make the Nile tilapia a good model to identify key genes responsible for MT-induced masculinization. In the present study, utilizing three male pathway gene mutant lines of Nile tilapia, including *amhy*, *dmrt1*, and *gsdf*, we investigated the molecular mechanism underlying MT-induced female-to-male sex reversal. Our findings suggested that MT-induced masculinization was dependent on *dmrt1* but not *amhy* and *gsdf*. MT directly inhibited early steroidogenesis independent of these three genes. However, MT-suppressed germ cell proliferation depends on *dmrt1*. Overall, we first demonstrated that exogenous MT-induced sex reversal depends on the activation of *dmrt1* and highlighted the indispensable role of *dmrt1* in teleost testis development.

## 2. Materials and Methods

### 2.1. Nile tilapia Culture and Genetic Sex Identification

Nile tilapia (*O. niloticus*) was cultured in freshwater tanks at approximately 26 °C under a natural photoperiod. The experimental fish were fed three times a day with commercial dry food (Shengsuo, Shandong, China). In our cultured conditions, all-XX and all-XY Nile tilapia develop as females and males, respectively. Genomic DNA was extracted from tail fins using the phenol/chloroform method, and the genetic sex of XX and XY fish was determined by a DNA marker developed previously [28]. Animal experiments were conducted following the Guidelines for Care and Use of Laboratory Animals approved by the Southwest University Animal Experimentation Committee.

### 2.2. Nile tilapia Mutant Lines

Three Nile tilapia mutant lines, including *amhy*, *gsdf*, and *dmrt1*, were established in our previous studies [24,25,26]. Specifically, the Nile tilapia mutants used in this study were produced by the following mating strategies: XY *amhy*^−^ mutants were produced by mating XX pseudo-males with XY *amhy*^−^ neo-females (producing functional eggs); XX/XY *gsdf*^−/−^ mutants were generated by crossing XY *gsdf*^+/−^ males with XX *gsdf*^+/−^ females; and XX/XY *dmrt1*^−/−^ mutants were produced by crossing XY *dmrt1*^+/−^ males with XX *dmrt1*^+/−^ females (Supplementary Figure S2). All primers used for sexing and genotyping are listed in Table S1.

### 2.3. Steroid Treatment

For the treatment group, 17α-methyltestosterone (MT, Sigma, St. Louis, MO, USA) was dissolved in 95% ethanol. It was supplemented with the commercial fish diet at a final concentration of 50 μg/g. Only 95% ethanol was added to the diet in the control group. Approximately 60 newly hatched Nile tilapia fry of each genotype were placed in different water tanks for the treatment and control groups. MT treatment was conducted from 5 to 30 dah (days after hatching). Subsequently, the treated fish were fed with a normal diet until 60 dah and then underwent histological observation and gene expression analysis. For the co-treatment with MT and fadrozole, fadrozole (Sigma, St. Louis, MO, USA) was added to the diet at the same time with a final concentration of 200 μg/g, and the treatment was extended to 50 dah.

### 2.4. Histological Observation and Immunofluorescence

The Nile tilapia gonads were sampled at the indicated time and fixed in Bouin’s solution overnight at room temperature. After dehydration, the gonads were embedded in paraffin, and cross-sections of 5 μm were obtained. For histological observation, gonad sections were stained with hematoxylin and eosin (H&E). Images were captured using an Olympus BX53F microscope (Olympus, Shinjuku, Tokyo, Japan). For immunofluorescence (IF), the sections were rehydrated and blocked in 5% fetal bovine serum (FBS)/PBS for 30 min at 37 °C and then incubated with primary antibodies in 5% BSA/PBS overnight at 4 °C. The primary antibodies used were Cyp19a1a (1:2000), 42Sp50 (1:500), Vasa (1:1000), Cyp11c1 (1:1000), Gsdf (1:1000), Cyp17a2 (1:1000), Star2 (1:1000), and Dmrt1 (1:500). The specificity of antibodies has been tested previously [26,29,30,31]. After removing the primary antibodies and washing with PBS buffer, the sections were incubated with Goat anti-rabbit Alexa Fluor 488 or 594 conjugated secondary antibodies (1:500, Thermo Fisher, Waltham, MA, USA) at room temperature for 1 h. The nuclei were counterstained with 4′,6-diamidino-2-phenylindole (DAPI) (1:1000, Sigma, St. Louis, MO, USA). The images were captured using a laser confocal microscope (Olympus FV3000, Shinjuku, Tokyo, Japan).

### 2.5. RNA Isolation, Transcriptome Sequencing, and Real-Time PCR Verification

Total RNA was extracted from the gonads of WT XX, WT XY, MT-treated XX, and MT-treated XX/XY *dmrt1*^−/−^ fish at 60 dah using RNAiso Plus (Takara, Kusatsu, Shiga, Japan). Half of the extracted RNA was used for transcriptome sequencing, while the other half was used for reverse transcription and subsequent quantitative real-time PCR. For transcriptome sequencing, the RNAs were subjected to 2 × 100 bp paired-end sequencing using the HiSeq 2000 platform (Illumina). Clean reads from each library were aligned to the reference genome (O_niloticus_UMD_NMBU GCA_001858045.3, http://asia.ensembl.org/Oreochromis_niloticus/Info/Index, 16 May 2023) using Tophat with default parameters. Gene expression levels were calculated using the fragments per kilobase of exon per million fragments mapped (FPKM) method. With Cuffmerge, the transcripts were combined using a reference annotation (*O. niloticus*: Orenil1.0.78.gtf, downloaded from Ensembl). Differential expression analysis was performed using Cuffdiff. The FPKM values of genes related to somatic and germ cell development were clustered using the TBtools software (v2.028) [32].

The cDNAs were synthesized using a PrimeScript^TM^ RT Reagent Kit with gDNA Eraser (Takara, Kusatsu, Shiga, Japan) according to the manufacturer’s instructions. Real-time PCR was performed on a 7500 Fast Real-Time PCR system (Applied Biosystems, Waltham, MA, USA) using Fast SYBR Green Master Mix (Takara, Kusatsu, Shiga, Japan), with *gapdh*, *β*-*actin*, and *eef1a1* as internal controls. The mRNA transcript abundance was calculated using the formula R = 2^−∆∆Ct^, as described previously [33]. At least three samples were analyzed for each genotype. Primers used for real-time PCR are listed in Table S1.

### 2.6. Whole-Mount Gonad Immunofluorescence

The Nile tilapia fry was dissected, and all internal organs were removed, leaving only the gonad attached to the coelomic epithelium. The head was then removed for DNA extraction. After sexing with a previously established DNA marker and genotyping, the body trunk containing the gonad was fixed in 4% PFA overnight at 4 °C and later dehydrated in methanol and stored at −20 °C until use. Prior to immunostaining, the fixed samples were rehydrated and permeabilized with 100% acetone for 10 min at 37 °C. After washing with PBS buffer, the samples were blocked in 5% FBS/PBS for 30 min at 37 °C. After removing the blocking buffer, the samples were incubated with primary antibodies including Cyp19a1a (1:2000), Cyp17a2 (1:1000), Star2 (1:1000), Vasa (1:1000), and Dmrt1 (1:500) overnight at 4 °C. After removing the primary antibodies and washing with PBS buffer, the samples were incubated with Goat anti-rabbit Alexa Fluor 488 or 594 conjugated secondary antibodies (1:500, Thermo Fisher, Waltham, MA, USA) at room temperature for 1 h. Finally, the samples were mounted on a glass slide, and images were captured using a laser confocal microscope (Olympus FV3000, Shinjuku, Tokyo, Japan).

### 2.7. Luciferase Assay

The Nile tilapia *cyp19a1a* promoter (–2346 bp) in a pGL3-basic vector and coding sequences of *sf-1*, *arα*, and *arβ* in a pcDNA3.1 vector were constructed in previous studies [15,34]. The culture of HEK293 cells, the transient transfections, and the luciferase assays were performed as previously reported [34]. Briefly, HEK293 cells were cultured in DMEM supplemented with 10% FBS, penicillin (100 IU/mL), and streptomycin (100 μg/mL) at 37 °C in a humidified cell incubator (Thermo Fisher, Waltham, MA, USA) containing 5% CO_2_. Transient transfections of 100 ng Renilla luciferase-expressing vector (pRL-TK) and 400 ng pGL3-*cyp19a1a* were performed at 60% confluence with lipofectamine 2000 (Thermo Fisher, Waltham, MA, USA). The cells were co-transfected with or without 100 ng pcDNA3.1-*sf-1*, -*arα*, and -*arβ* vectors. MT was added to the cultured cells 4 h after vector transfection at final concentrations of 1 × 10^−4^ M and 2 × 10^−4^ M. Complete inhibition of aromatase activity has been shown at these concentrations [35]. The transfection efficiency was monitored using the pRL-TK vector as the internal control. Luciferase activity was quantified using the Dual-Luciferase Reporter Assay System (Promega, Madison, WI, USA) and the Luminoskan ascent luminometer (Thermo Fisher, Waltham, MA, USA). The relative luciferase activity was determined by dividing the firefly luciferase activity by the Renilla luciferase activity.

### 2.8. EdU Incorporation and Germ Cell Proliferation Analysis

The Click-iT EdU Cell Proliferation Kit (Invitrogen, Waltham, MA, USA) was used for analyzing germ cell proliferation. For EdU (5-ethynyl-2′-deoxyuridine) incorporation, it was dissolved in water at a concentration of 20 mg/mL and stored at −20 °C before use. Approximately 40 newly hatched Nile tilapia fry of each genotype were placed in a small glass water tank containing 2 L of water, to which EdU was added at a final concentration of 100 mg/L. The water tank was kept away from light, and the EdU-containing water was replaced every two days. For the control group, only EdU was added to the water, and they were fed with a normal diet. For the EdU and MT co-treatment group, MT was added to the diet at a final concentration of 50 μg/g. The treatment lasted for 10 days, from 5 dah to 15 dah. EdU staining was performed in accordance with the manufacturer’s instructions. Briefly, after rehydration and permeabilization, the fixed samples were washed three times with PBS buffer and then incubated with a freshly prepared EdU detection mix (containing CuSO_4_, Alexa Fluor azide, 1X Click-iT reaction buffer, and reaction buffer additive) for 30 min at room temperature, avoiding the light. After removal of the detection mix, the samples were washed again with PBS buffer and then blocked in 5% FBS/PBS for 30 min at room temperature. Finally, the samples were stained with Vasa primary antibody and Alexa Fluor 488-conjugated secondary antibody (Thermo Fisher, Waltham, MA, USA). The number of germ cells was counted after Vasa antibody staining. At least three samples for each genotype were analyzed. Images were taken using a laser confocal microscope (Olympus FV3000, Shinjuku, Tokyo, Japan).

### 2.9. Data Analysis

All the experiments in this study were conducted at least three times. Data were expressed as the mean ± SD, using GraphPad Prism 8 software (v8.0.1). The significance of differences between groups was calculated using one-way ANOVA, followed by Tukey’s test for multiple comparisons. For all statistical analyses, *p* < 0.05 indicates a statistically significant difference.

## 3. Results

### 3.1. amhy and gsdf Mutants But Not dmrt1 Mutants Were Masculinized by MT Treatment

**Figure 1:**
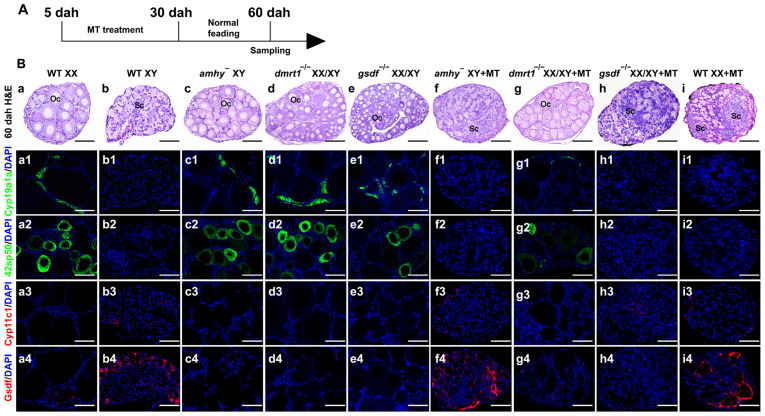
17α-Methyltestosterone treatment masculinized *amhy* and *gsdf* mutants but not *dmrt1* mutants. (**A**) Schematic representation of MT treatment and sampling. (**B**) Gonad histological analysis of WT XX, WT XY, XY *amhy*^−^, XX/XY *dmrt1*^−/−^, XX/XY *gsdf*^−/−^, and MT-treated WT XX, -XY *amhy*^−^, -XX/XY *dmrt1*^−/−^, -XX/XY *gsdf*^−/−^ fish using hematoxylin and eosin (H&E) staining and gene expression analysis by immunofluorescence (IF) at 60 dah. After MT treatment, testis developed in XY *amhy*^−^ and XX/XY *gsdf*^−/−^ mutants, whereas ovary developed in XX/XY *dmrt1*^−/−^ mutants. Expressions of female marker Cyp19a1a (**B**, **a1**–**i1**), oocyte marker 42Sp50 (**B**, **a2**–**i2**), and male markers Cyp11c1 (**B**, **a3**–**i3**) and Gsdf (**B**, **a4**–**i4**) were analyzed by IF at 60 dah. MT-treated *dmrt1* mutant ovary showed similar expression patterns of these markers with WT XX fish. DAPI (4′,6-diamidino-2-phenylindole) was used to counterstained the nuclei. MT, 17α-methyltestosterone; dah, days after hatching; WT, wild type; Oc, oocyte; Sc, spermatocyte; scale bars in (**B**) 50 μm.

To determine the male pathway gene required for MT-induced masculinization in Nile tilapia, we performed MT treatment from 5 dah to 30 dah on F2 fish siblings of three mutant lines, including *amhy*, *dmrt1*, and *gsdf* (Figure 1A). After MT treatment and 30 days of normal feeding, the gonadal phenotype was examined by H&E staining at 60 dah. Consistent with our previous studies [24,25,26], the gonads of XY *amhy*^−^, XX/XY *dmrt1*^−/−^, and XX/XY *gsdf*^−/−^ mutants developed as ovaries with previtellogenic oocytes, similar to those of the wild type (WT) XX fish. Whereas the gonads of MT-treated XY *amhy*^−^, MT-treated XX/XY *gsdf*^−/−^, and MT-treated XX fish developed as testes with spermatogonia and spermatocytes, similar to those of the WT XY fish. In contrast, the gonads of MT-treated XX/XY *dmrt1*^−/−^ mutants still developed as ovaries with previtellogenic oocytes (Figure 1B(a–i)). The masculinization rate of different genotypes by MT treatment was listed in Table S2. Gene expression analysis by immunofluorescence (IF) showed that the female marker Cyp19a1a and the oocyte marker 42Sp50 were normally expressed in the gonads of MT-treated XX/XY *dmrt1*^−/−^ mutants, same as the untreated mutants and WT XX fish (Figure 1B(a1–i2)). On the other hand, the expression of male-specific Cyp11c1 was detected in the gonads of MT-treated XY *amhy*^−^, MT-treated XX/XY *gsdf*^−/−^, and MT-treated XX fish, as well as the WT XY fish. Another male-biased gene, Gsdf, was also significantly up-regulated in the gonads of MT-treated XY *amhy*^−^ mutants and MT-treated XX fish, as well as in the WT XY fish. Conversely, the expression of Cyp11c1 and Gsdf in the gonads of MT-treated XX/XY *dmrt1*^−/−^ mutants was similar to that of the WT XX fish (Figure 1B(a3–i4)). Although the gonads of MT-treated XY *amhy*^−^, -XX/XY *gsdf*^−/−^, and -XX fish appear slightly abnormal in structure at this time, they all develop into normal testes with different stages of spermatogenic cells at 6 months (Supplementary Figure S3). These results indicate that MT-induced female-to-male sex reversal depends on *dmrt1* and not *amhy* and *gsdf*.

### 3.2. Gonads of dmrt1 Mutants Retained Female Identity after MT Treatment

**Figure 2:**
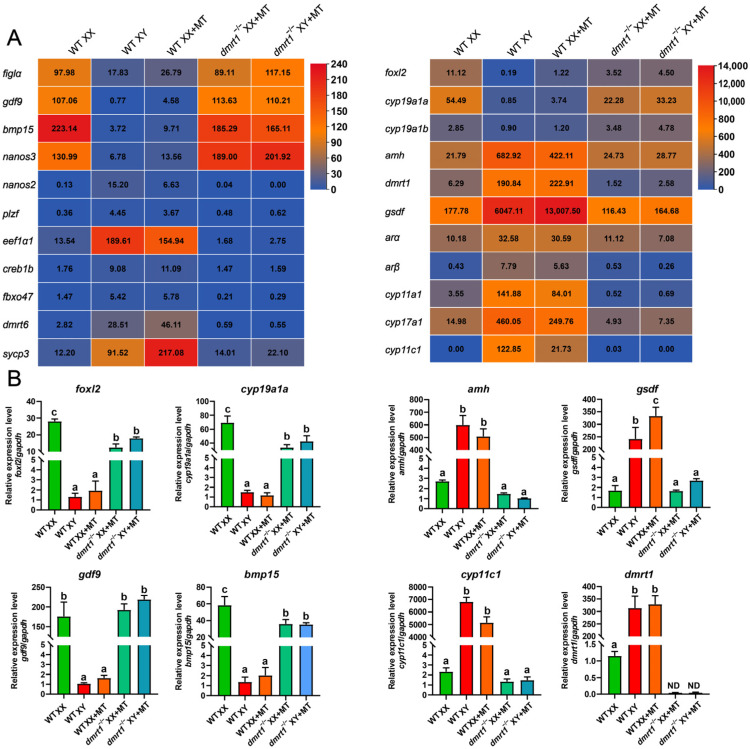
Gonad transcriptome data of MT-treated *dmrt1* mutants. (**A**) Transcriptome analysis of gene expression (FPKM value) in WT XX, WT XY, MT-treated XX, and MT-treated XX/XY *dmrt1*^−/−^ gonads at 60 dah. Gene expression patterns of MT-treated *dmrt1* mutants were similar to those of the WT XX controls. (**B**) Real-time PCR verification of female markers *foxl2*, *cyp19a1a*, *gdf9*, and *bmp15* and male markers *amh*, *gsdf*, *cyp11c1*, and *dmrt1* in WT XX, WT XY, MT-treated XX, and MT-treated XX/XY *dmrt1*^−/−^ fish gonads. Expression was normalized to *gapdh*. Data were expressed as the mean ± SD. Different letters above the error bars indicate statistical differences at *p* < 0.05 as determined by one-way ANOVA followed by Tukey’s test. WT, wild type; ND, not detected; MT, 17α-methyltestosterone.

To further characterize the gonadal gene expression profiles of MT-treated *dmrt1* mutants, we performed transcriptome analysis at 60 dah following MT treatment. Comparative analysis of genes related to gonadal somatic and germ cell development showed that *dmrt1*^−/−^ mutants maintained a typical female gene expression pattern (Figure 2A). The expression of genes related to male sex differentiation, such as *amh*, *gsdf*, *dmrt1*, and two *ars*, was highly up-regulated in MT-treated XX fish, comparable to that of the WT XY fish. In contrast, the expression of these genes remained low in MT-treated *dmrt1*^−/−^ mutants, similar to that of the WT XX fish. Additionally, genes essential for female differentiation in MT-treated *dmrt1*^−/−^ mutants, including *foxl2*, *cyp19a1a*, and *cyp19a1b*, were expressed similarly to those of the WT XX fish. Consistently, the oocyte markers *bmp15* and *gdf9* were expressed at high levels in MT-treated *dmrt1*^−/−^ mutants, indicating the adoption of a female fate after MT treatment in germ cells lacking *dmrt1*. As expected, the spermatogonia markers *nanos2* and *eef1α1* and the spermatocyte marker *dmrt6* were highly expressed in MT-treated XX fish and WT XY fish but remained low in MT-treated *dmrt1*^−/−^ mutants and WT XX fish. Interestingly, *sycp3*, a meiosis marker gene, was significantly up-regulated in MT-treated XX gonads compared with WT XY gonads. In addition, the expression of these male- or female-biased genes was further confirmed by real-time PCR analysis (Figure 2B, Supplementary Figure S4). These results indicate that MT treatment failed to induce male fate in gonad cells in the absence of *dmrt1*.

### 3.3. Co-Treatment with MT and AI Failed to Induce Sex Reversal in dmrt1 Mutants

**Figure 3:**
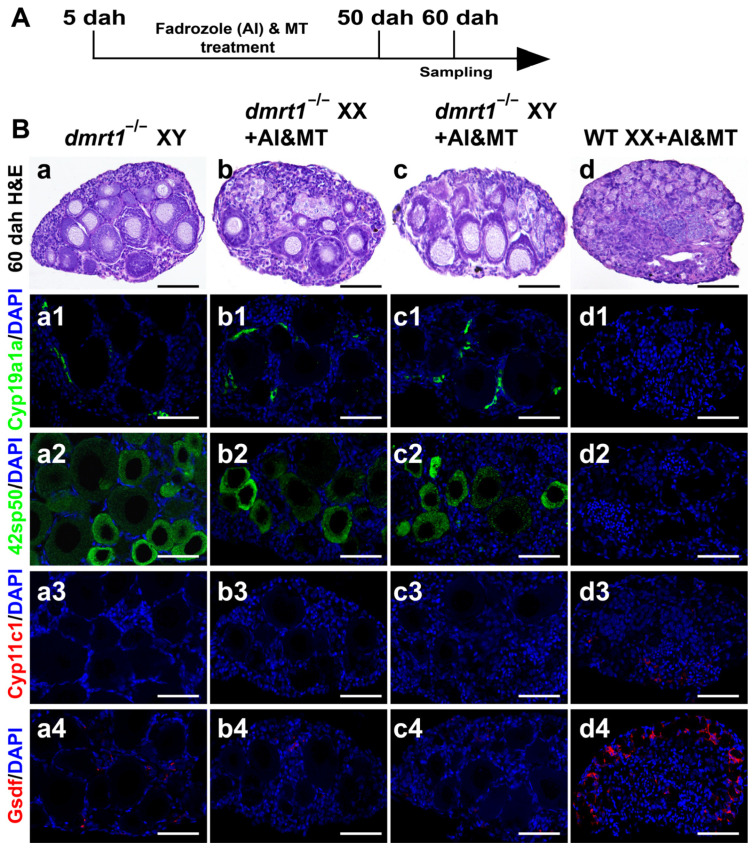
Simultaneously, administration with AI and MT failed to induce female-to-male sex reversal in *dmrt1^−/−^* mutants. (**A**) Schematic representation of AI&MT co-treatment and sampling. (**B**, **a**–**d**) Histological examination of *dmrt1*^−/−^ XY, AI&MT-treated XX, and AI&MT-treated *dmrt1*^−/−^ XX/XY gonads at 60 dah by H&E. (**B**, **a1**–**d4**) Immunofluorescence analysis of female marker Cyp19a1a, oocyte marker 42Sp50, and male markers Cyp11c1 and Gsdf in *dmrt1*^−/−^ XY, AI&MT-treated XX, and AI&MT-treated *dmrt1*^−/−^ XX/XY gonads at 60 dah. Ovaries developed after simultaneous treatment with AI and MT in *dmrt1* mutants. DAPI was used to counterstain the nuclei. WT, wild type; AI, aromatase inhibitor, fadrozole; MT, 17α-methyltestosterone; dah, days after hatching; scale bars in (**B**) 50 μm.

Previously, we showed that treatment of XX/XY *dmrt1*^−/−^ mutants with the aromatase inhibitor (AI) fadrozole resulted in ovarian development [26]. To determine whether treatment of the *dmrt1*^−/−^ mutants with a combination of AI and MT (AI&MT) could induce testis development, we performed co-treatment on *dmrt1* mutants with an extended treatment time of 5 to 50 dah (Figure 3A). Histological examination showed that the gonads of the AI&MT-treated XX fish developed into normal testes, whereas the gonads of the AI&MT-treated XX/XY *dmrt1*^−/−^ mutants all developed into ovaries, similar to the XY *dmrt1*^−/−^ mutants at 60 dah (Figure 3B(a–d)). The masculinization rate of different genotypes by AI&MT treatment is listed in Table S2. IF staining with Cyp19a1a and 42Sp50 antibodies revealed the presence of these two proteins in the gonads of the AI&MT-treated XX/XY *dmrt1*^−/−^ mutants and XY *dmrt1*^−/−^ mutants, as well as their absence in the gonads of AI&MT-treated XX fish (Figure 3B(a1–d2)). In contrast, IF staining with Cyp11c1 showed its exclusive expression in the gonads of AI&MT-treated XX fish (Figure 3B(a3–d3)). Similarly, another testis-biased gene, Gsdf, also showed a much higher expression in the AI&MT-treated XX fish than in the other three genotypes (Figure 3B(a4–d4)). These results show that combined treatment with MT and AI could not induce testis development without the *dmrt1* gene.

### 3.4. MT Treatment Inhibited Early Steroidogenesis Independent of Male Pathway Genes

**Figure 4:**
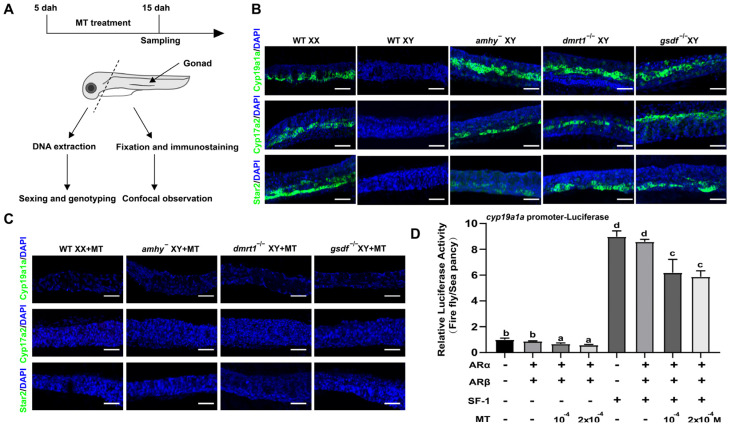
MT treatment directly inhibits steroidogenic enzyme expression independent of *amhy*, *dmrt1*, and *gsdf*. (**A**) Schematic representation of MT treatment and sampling time. (**B**) Expression of steroidogenic enzymes by whole-mount gonad immunofluorescence at 15 dah. Expression of Star2, Cyp17a2, and Cyp19a1a was detected in the gonads of XY *amhy*^−^, XY *gsdf*^−/−^, XY *dmrt1*^−/−^ mutants, and WT XX fish, but not in WT XY fish. (**C**) Expression of Star2, Cyp17a2, and Cyp19a1a at 15 dah after 10 days of MT treatment. MT treatment completely inhibited the expression of Star2, Cyp17a2, and Cyp19a1asteroidogenic enzymes in WT XX fish, XY *amhy*^−^, XY *gsdf*^−/−^, and XY *dmrt1*^−/−^ mutants. (**D**) MT treatment suppressed basal and Sf-1-activated *cyp19a1a* promoter activity in HEK293 cells in the presence of two *ar* receptors. Data were expressed as mean ± SD. Different letters above the error bars indicate statistical differences at *p* < 0.05 as determined by one-way ANOVA followed by Tukey’s test. DAPI was used to counterstain the nuclei. WT, wild type; MT, 17α-methyltestosterone; dah, days after hatching; scale bars in (**B**,**C**) 20 μm.

In Nile tilapia, the gonadal steroidogenic enzymes were expressed in the female gonad but not in the male gonad at the early developmental stage [36,37]. Inhibition of these steroidogenic enzymes by MT treatment has been reported previously [15]. To test whether this inhibition requires the presence of male pathway genes, we performed whole-mount gonad IF to examine steroidogenic enzyme (Star2, Cyp17a2, and Cyp19a1a) expression at 15 dah, the time for MT treatment (Figure 4A). The IF results demonstrated that the gonads from XY *amhy*^−^, XY *dmrt1*^−/−^, and XY *gsdf*^−/−^ mutants expressed the steroidogenic enzymes (Star2, Cyp17a2, Cyp19a1a) at levels similar to those of the WT XX gonads at 15 dah, while these steroidogenic enzymes were not expressed in the WT XY gonads (Figure 4B). In the MT treatment group, the expression of these three steroidogenic enzymes was completely eliminated in the MT-treated XX gonads and -XY *amhy*^−^, -XY *dmrt1*^−/−^, and -XY *gsdf*^−/−^ mutants (Figure 4C). Additionally, luciferase analysis showed that MT treatment exhibited a direct inhibition of *cyp19a1a* promoter activity in cultured HEK293 cells in the presence of androgen receptors (*arα* and *arβ*) (Figure 4D). These results indicate that MT inhibits the expression of steroidogenic enzymes independent of the three male pathway genes (*amhy*, *dmrt1*, *gsdf*).

### 3.5. Dmrt1 Is Required for MT-Induced Inhibition of Germ Cell Proliferation

**Figure 5:**
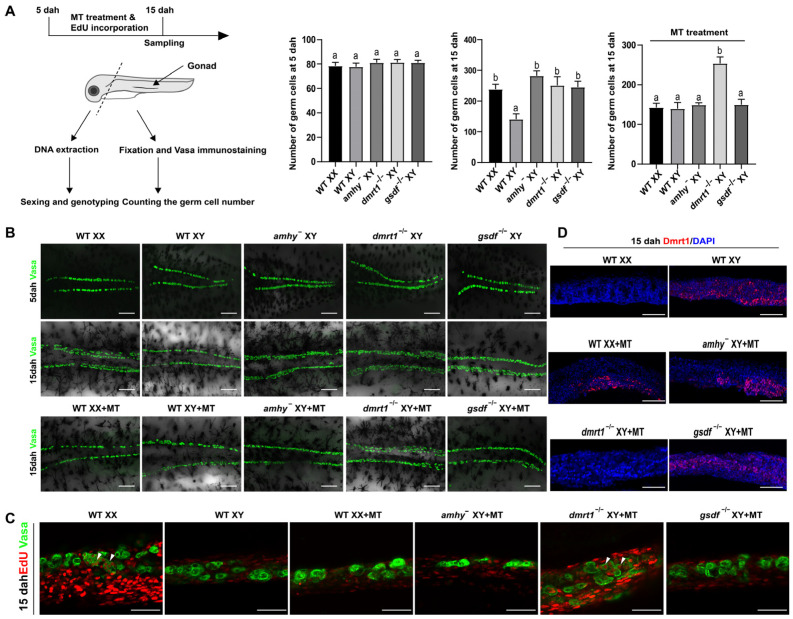
Inhibition of germ cell proliferation by MT depends on Dmrt1. (**A**) Schematic representation of MT treatment with EdU incorporation and sampling. (**B**) Whole-mount gonad immunofluorescence with Vasa antibody and germ cell number counting at 5 and 15 dah. The similar number of germ cells in WT XX, WT XY, XY *amhy*^−^, XY *gsdf*^−/−^, and XY *dmrt1*^−/−^ fish was observed at 5 dah, while the number of germ cells in WT XX, XY *amhy*^−^, XY *gsdf*^−/−^, and XY *dmrt1*^−/−^ fish was significantly higher than that of the WT XY fish at 15 dah. The germ cell number of MT-treated XX, -XY *amhy*^−^, and -XY *gsdf*^−/−^ fish was comparable to that of the WT XY fish, while the germ cell number of MT-treated XY *dmrt1*^−/−^ fish was comparable to that of WT XX fish. Data were expressed as mean ± SD. Different letters above the error bars indicate statistical differences at *p* < 0.05 as determined by one-way ANOVA followed by Tukey’s test. (**C**) EdU (red)/Vasa (green) double staining was performed to show germ cell proliferation in MT-treated XY *dmrt1*^−/−^ fish, same as the WT XX fish. (**D**) Expression of Dmrt1 by whole-mount gonad immunofluorescence at 15 dah. Expression of Dmrt1 was up-regulated in MT-treated XX fish, MT-treated XY *amhy*^−^, and -XY *gsdf*^−/−^ mutants compared to WT XX fish at 15 dah. DAPI was used to counterstain the nuclei. WT, wild type; MT, 17α-methyltestosterone; EdU, 5-ethynyl-2-deoxyuridine; dah, days after hatching; scale bars in (**B**) 50 μm, (**C**,**D**) 20 μm.

Germ cells proliferate earlier in females than males in Nile tilapia [22]. To detect the effects of MT treatment on early germ cell mitotic activity, we performed MT treatment accompanied by EdU incorporation analysis from 5 to 15 dah (Figure 5A). Germ cell counting after Vasa staining revealed a similar germ cell number among WT XX, WT XY fish, XY *amhy*^−^, XY *gsdf*^−/−^, and XY *dmrt1*^−/−^ mutants at 5 dah. Later on, the number of germ cells in the WT XX fish and the three types of mutants was significantly higher than that of the WT XY fish at 15 dah (Figure 5B). After MT treatment, the number of germ cells in MT-treated XX fish, -XY *amhy*^−^, and -XY *gsdf*^−/−^ mutants reduced to a similar level as the WT XY fish at 15 dah. However, the number of germ cells in MT-treated XY *dmrt1*^−/−^ mutants remains unchanged, similar to WT XX fish at this time point (Figure 5B). Co-staining with EdU and the Vasa antibody detected proliferating germ cells in WT XX and MT-treated XY *dmrt1*^−/−^ mutant gonads, whereas no EdU-positive germ cells were present in MT-treated XX, -XY *amhy*^−^, and -XY *gsdf*^−/−^ mutant gonads (Figure 5C). To further test whether Dmrt1 is associated with this inhibition of germ cell proliferation, we examined the Dmrt1 expression during MT treatment. The results showed that Dmrt1 was expressed in the gonads of MT-treated XX fish, -XY *amhy*^−^, and -XY *gsdf*^−/−^ mutants, same as WT XY fish, whereas no Dmrt1 signal was detected in the gonads of WT XX fish or MT-treated XY *dmrt1*^−/−^ mutants (Figure 5D). These results suggest that MT-induced inhibition of germ cell proliferation relies on *dmrt1*.

## 4. Discussion

Although endogenous androgens are not involved in sex determination in fish, exogenous androgens treatment can induce female-to-male sex reversal during gonadal sex differentiation in a number of fish species [3]. A recent report in medaka showed that androgens are the key factor involved in temperature-induced masculinization because high temperatures failed to induce female-to-male sex reversal when androgen action is inhibited [38]. However, to date, the molecular mechanism by which exogenous androgens induce sex reversal is largely unknown. In this study, we showed that 17α-methyltestosterone (MT) induced masculinization of WT XX Nile tilapia as well as *amhy* and *gsdf* mutants, but not *dmrt1* mutants, suggesting a critical role of *dmrt1* in MT-induced masculinization. MT has been shown to bind to and act through the androgen receptor (*ar*) [39,40]. A previous study in Nile tilapia showed that the expression of steroidogenic enzymes (Cyp11a1, 3β-HSD, and Cyp19a1a) was inhibited by MT [15]. Our results also showed that the steroidogenic enzyme (Star2, Cyp17a2, and Cyp19a1a) expression was repressed by MT, even in the absence of three male pathway genes (*amhy*, *dmrt1*, and *gsdf*). Consistently, a luciferase analysis showed the direct suppression of *cyp19a1a* promoter activity by MT through two *ars* (*arα* and *arβ*), which provide evidence to support the in vivo data. The expression of two *ars* in the MT-treated Nile tilapia XX gonad was increased to similar levels as in the XY testis. This has also been shown in the orange-spotted grouper (*Epinephelus coioides*), where *arβ* expression was up-regulated significantly after two weeks of MT implantation [41]. Combined with the fact that two androgen receptors are expressed in the XX Nile tilapia gonad at 5 and 30 dah [37], we concluded that MT acts through *ar* to directly block steroidogenic enzyme expression and estrogen production and that this process is independent of the male pathway genes (*amhy*, *dmrt1*, *gsdf*).

Recently, we reported that once *dmrt1* was mutated, the gonad could not be reversed to functional testis by mutating any female pathway gene (*foxl2*, *foxl3*, and *cyp19a1a/b*) or fadrozole treatment in Nile tilapia [42]. Consistently, our study showed that MT treatment up-regulated *dmrt1* expression, leading to testis development in *amhy* and *gsdf* mutants, whereas treatment with MT or a combination of MT and AI failed to rescue the male-to-female sex reversal in *dmrt1* mutant Nile tilapia. Similar results were shown in the spotted scat (*Scatophagus argus*), where MT or AI treatment failed to induce female-to-male sex reversal due to the absence of a functional *dmrt1* copy in XX individuals [43]. In a report of northern medaka, MT induced Gsdf expression but not Dmrt1 directly in XX gonads to reverse sex [17]. However, this was tested only on the expression level, and no *gsdf* mutant model was used to demonstrate the role of *gsdf* in androgen-induced sex reversal. Nevertheless, evidence from other vertebrates showed the following: (1) In Z*^dmrt1^*^−^W chickens (*Gallus gallus*), fadrozole treatment cannot induce female-to-male sex reversal [44]. (2) In turtles (*Trachemys scripta*) with temperature-dependent sex determination, knockdown of *dmrt1* resulted in ovary development even at male-promoting temperature [45]. These findings indicate that Dmrt1 has a conserved and pivotal role in testis development and unique properties that make it irreplaceable [46].

In Nile tilapia, as in many other teleosts, germ cells proliferate and initiate meiosis earlier in females than in males [47]. In this study, MT treatment inhibited germ cell proliferation in WT XX Nile tilapia, *amhy* mutants, and *gsdf* mutants, but not in *dmrt1* mutants. The expression of Dmrt1 was induced in the MT-treated *amhy* and *gsdf* mutants, suggesting that MT treatment might inhibit germ cell proliferation via up-regulation of Dmrt1 in Nile tilapia. Studies in zebrafish showed that loss of *ar* resulted in decreased *dmrt1* expression [48]. Additionally, the sex-determining gene *dmrt1bY* in medaka was shown to induce mitosis arrest of PGCs in XY individuals [49]. Since *dmrt1bY* is a duplicate of the autosomal *dmrt1*, it is suggested that *dmrt1* might retain its inhibitory role and function under sex reversal conditions in XX females [50]. This was supported by high temperature and cortisol [50] and starvation [51]-induced masculinization experiments, in which *dmrt1* was shown to be required. In addition, Dmrt1 has been shown to be a dose-sensitive regulator of fetal germ cell proliferation via direct regulation of the pluripotency gene *Sox2* in mice [52]. Considering these evidences, we suggest that the control of germ cell proliferation via Dmrt1 might be a common phenomenon in fish.

## 5. Conclusions

**Figure 6:**
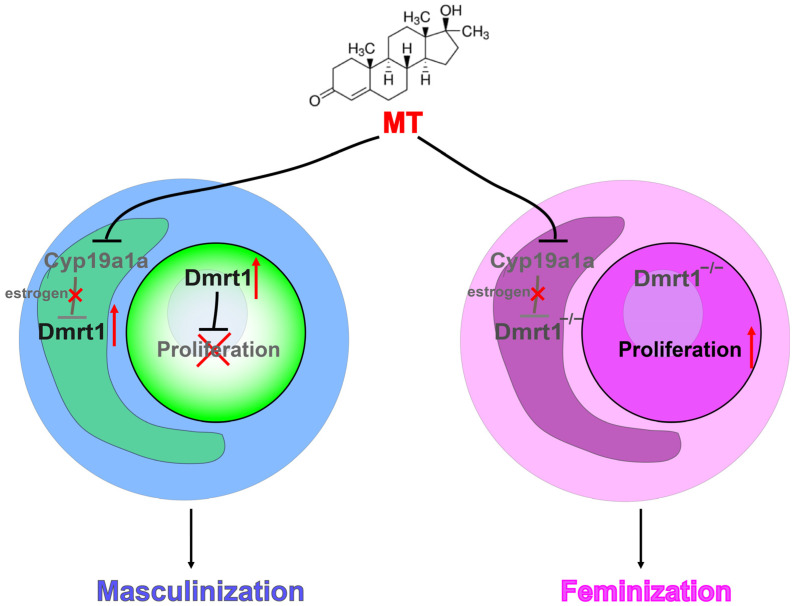
Indispensable role of Dmrt1 in MT-induced masculinization in Nile tilapia.

In conclusion, Nile tilapia *amhy* and *gsdf* mutants, but not *dmrt1* mutants, were successfully masculinized by the MT treatment. The MT treatment completely blocked early steroidogenic enzyme expression independent of *amhy*, *gsdf*, and *dmrt1*, while inhibition of germ cell proliferation by the MT treatment requires the presence of *dmrt1*. These results suggest that *dmrt1* is indispensable for MT-induced masculinization in Nile tilapia and that MT functions by inhibiting early steroid synthesis and activating *dmrt1* to promote testis development (Figure 6).

A proposed model for the role of Dmrt1 in MT-induced masculinization in Nile tilapia. In the presence of Dmrt1, MT treatment inhibits early steroidogenesis and activates Dmrt1 expression to block germ cell proliferation, thereby promoting testis development. In the absence of Dmrt1, MT directly inhibits early steroidogenesis but fails to induce masculinization.

## Data Availability

The sequenced transcriptome data have been deposited in the Short Read Archive of NCBI with the accession number PRJNA1017631.

## Supplementary Materials

The following supporting information can be downloaded at https://www.mdpi.com/article/10.3390/genes15091238/s1, Figure S1: Nile tilapia male pathway genes antagonize female pathway genes; Figure S2: Establishment of Nile tilapia mutants used in this study; Figure S3: Histological observation after MT treatment at 180 dah; Figure S4: Real-time PCR results using *β*-*actin* and *eef1a1* as internal controls; Table S1: Primers used in the present study; Table S2: Masculinization rate of WT XX fish and different mutants by MT treatment and AI&MT treatment.
