# Supplementary material for: dmrt1 Is Responsible for Androgen-Induced Masculinization in Nile Tilapia
Source: Genes (Basel). 2024 Sep 23;15(9):1238. doi: 10.3390/genes15091238 (PMC11431369; doi:10.3390/genes15091238)
Supplement: Supplementary file 1 [file genes-15-01238-s001.zip › genes-3155088-supplementary.pdf]

Supplemental figures and tables

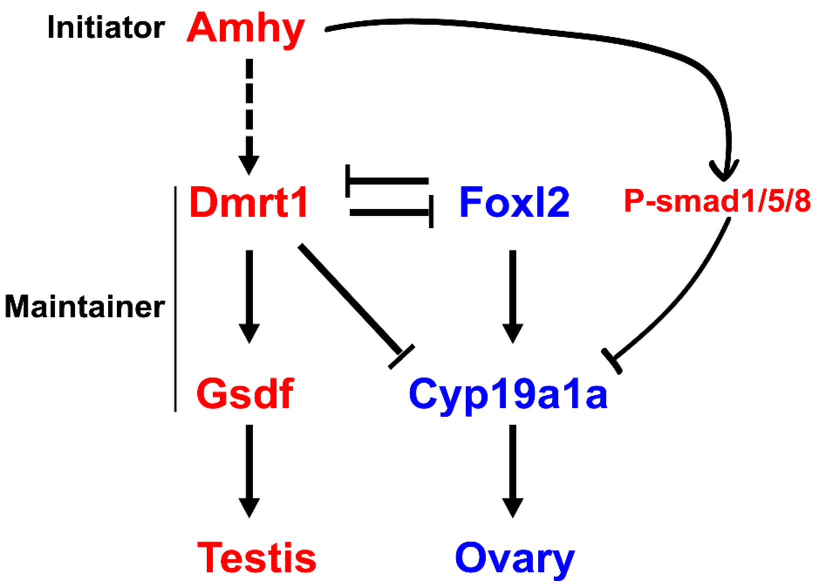

Figure S1. Nile tilapia male pathway genes antagonize female pathway genes.

In Nile tilapia, *amhy* serves as the male sex initiator to inhibit E2 production and activate downstream male sex maintainers *dmrt1* and *gsdf* to further antagonize female pathway genes to ensure testis development.

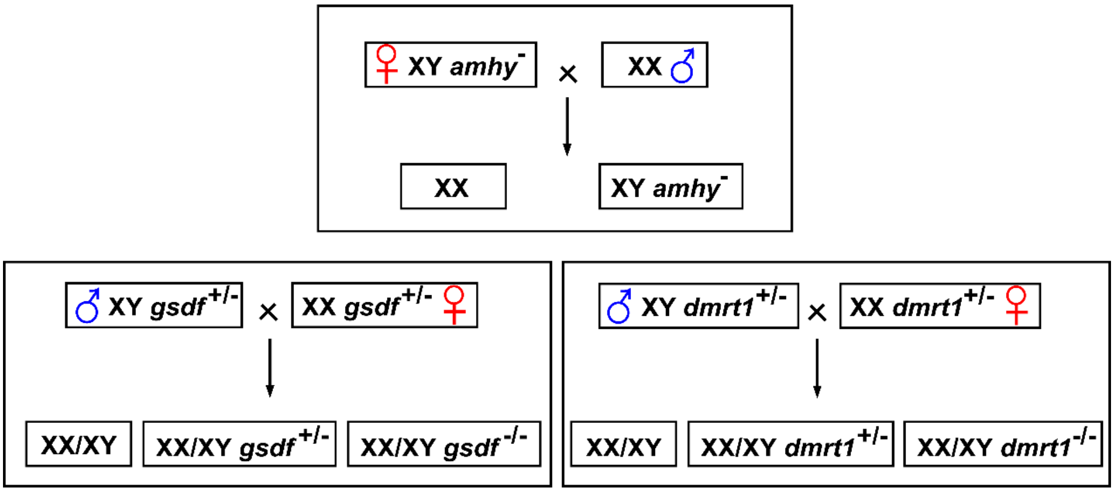

Figure S2. Establishment of Nile tilapia mutants used in this study.

XY *amhy*$^{-}$ mutants were produced by mating XX pseudo-males with XY *amhy*$^{-}$ neo-females (producing functional eggs); XX/XY *gsdf*$^{-/-}$ mutants were generated by crossing XY *gsdf*$^{+/-}$ males with XX *gsdf*$^{+/-}$ females; and XX/XY *dmrt1*$^{-/-}$ mutants were produced by crossing XY *dmrt1*$^{+/-}$ males with XX *dmrt1*$^{+/-}$ females.

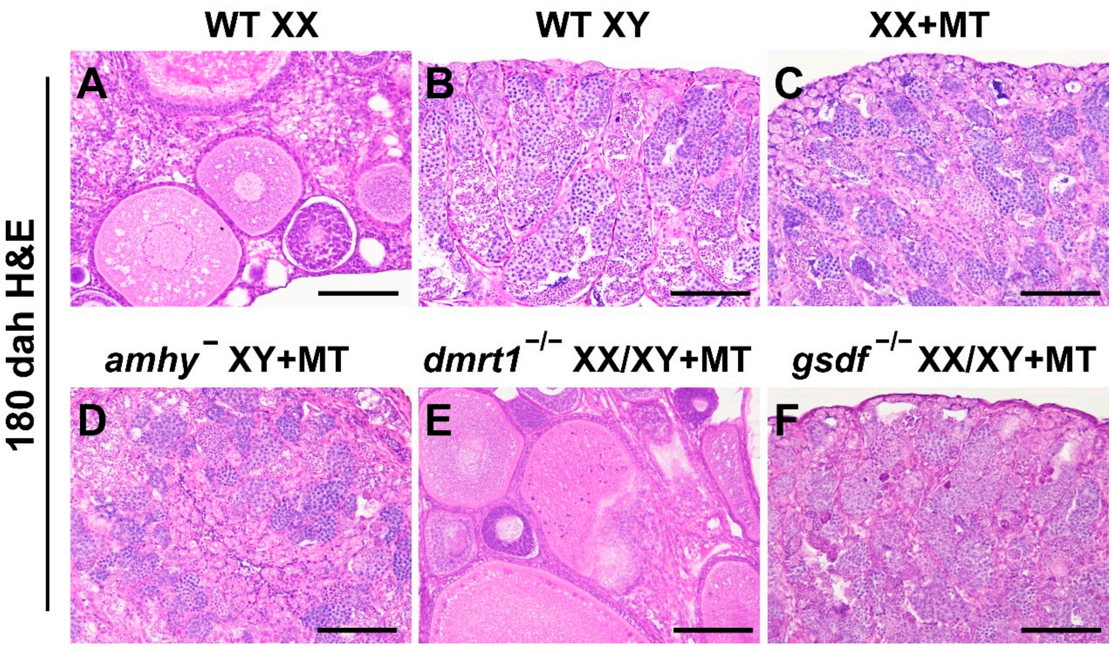

Figure S3. Histological observation after MT treatment at 180 dah.

Gonadal histological observation of WT XX, WT XY, and MT-treated WT XX, -XY *amhy*$^{-}$, -XX/XY *dmrt1*$^{-/-}$, - XX/XY *gsdf*$^{-/-}$ fish at 180 dah using hematoxylin and eosin (H&E) staining. Gonads of MT-treated WT XX, -XY *amhy*$^{-}$ and XX/XY *gsdf*$^{-/-}$ fish developed as testes, while gonads of MT-treated XX/XY *dmrt1*$^{-/-}$ fish developed as ovaries. WT, wild type; MT, 17α-methyltestosterone; dah, days after hatching; scale bar: 100 µm.

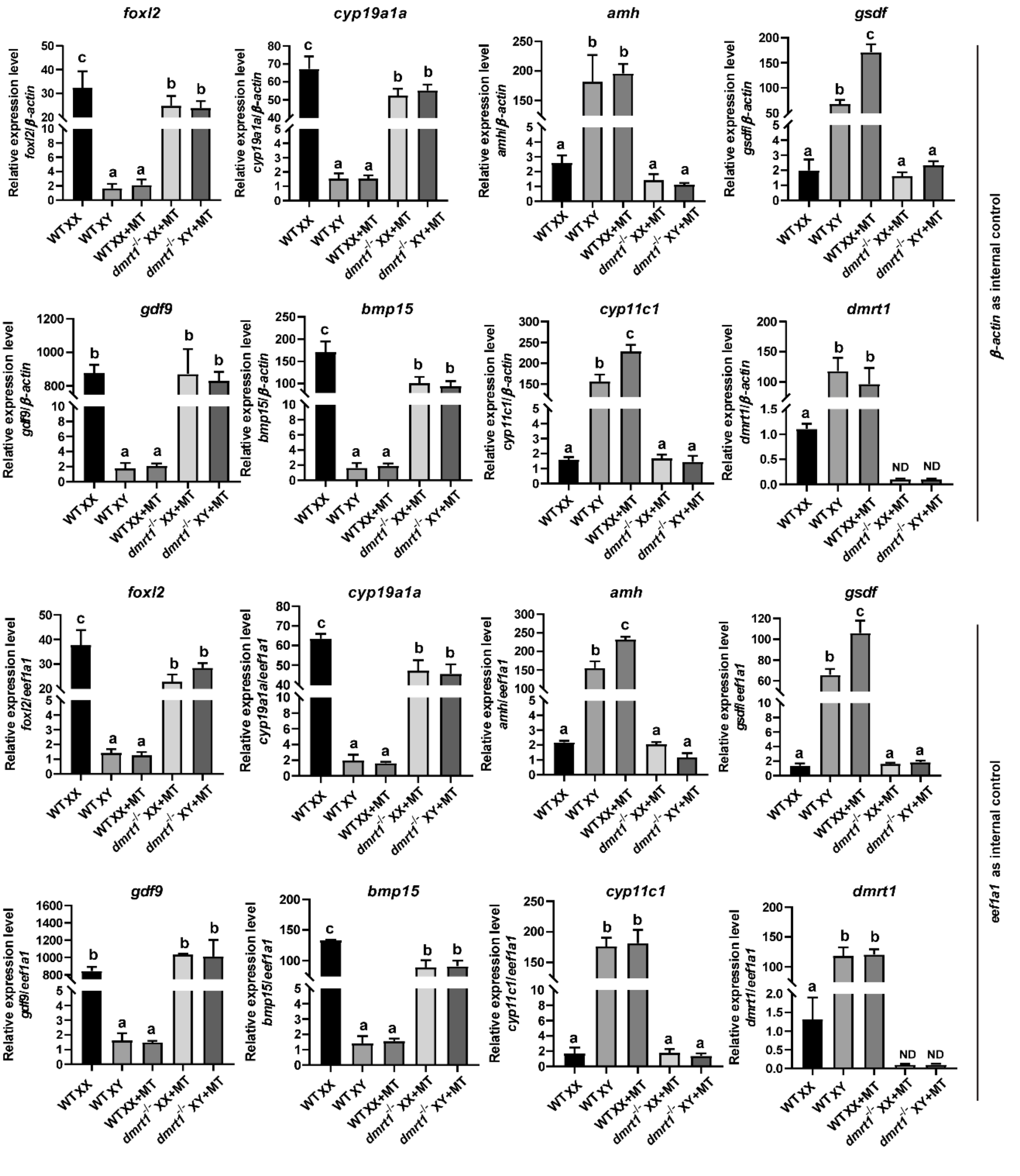

Figure S4. Real-time PCR results using *β-actin* and *eef1a1* as internal controls.

Real-time PCR verification of female markers *foxl2*, *cyp19a1a*, *gdf9*, *bmp15* and male markers *amh*, *gsdf*, *cyp11c1*, *dmrt1* in WT XX, WT XY, MT-treated XX, MT-treated XX/XY *dmrt1*$^{-/-}$ fish gonads. Expression was normalized to *β-actin* and *eef1a1*. Data were expressed as the mean ± SD, Different letters above the error bars indicate statistical differences at P<0.05 as determined by one-way ANOVA followed by Tukey test. WT, wild type; ND, not detected; MT, 17α-methyltestosterone.

Supplementary Table S1. Primers used in the present study.

Primer	Sequence	Purpose
foxl2-qPCR-F1	AAGAGGAGCCGGTTCAGGACAA	Real-time PCR
foxl2-qPCR-R1	GCTCTCCCGGATAGCCATGG	

cyp19a1a-qPCR-F1	GCCATCCAATCGATGGGATATC	
cyp19a1a-qPCR-R1	CTCAGATATGACAGAAGTGGGCG	
bmp15-qPCR-F1	ACGAGCAGAAAGCGGACCAGA	
bmp15-qPCR-R1	GCGGAGAAGAGCGAAGGTGAAC	
gdf9-qPCR-F1	GGAGTGTGGACGGGAGCA	
gdf9-qPCR-R1	GGCTGTAGGACAAATCCT	
amh-qPCR-F1	CACCCAGCTGCAGTACACGTAT	
amh-qPCR-R1	TCAAAGGTCAACGTGATTGTTCC	
gsdf-qPCR-F1	GCTACCTGCCGGTGCACT	
gsdf-qPCR-R1	AGCCTGGGACTGCTGGG	
cyp11c1-qPCR-F1	CGCTTCCAGCAGCTTCACA	
cyp11c1-qPCR-R1	CAGATGGCAGCATGATGT	
dmrt1-qPCR-F1	CGGCCCAGGTTGCTCTGAG	
dmrt1-qPCR-R1	CCAACTTCATTCTTGACCATCA	
β-actin-qPCR-F1	GGCATCACACCTTCTACAACGA	
β-actin-qPCR-R1	ACGCTCTGTCAGGATCTTCA	
eef1a1-qPCR-F1	CAAGTGCGGAGGAATCGA	
eef1a1-qPCR-R1	CGAACTTCCACAGAGCGATA	
gapdh-qPCR-F1	AAGCTCATTTCCTGGTAT	
gapdh-qPCR-R1	CCTTTGCTGATTTCCTTG	
amhy-KO-S-F	GAAAGGGGTGTTTTGGTGCTGGC	Mutants screening
amhy-KO-S-R	CAGGGTTTGCACCTGAGCTCTC	
dmrt1-KO-S-F	GCCAAAAAGCAGGGTGTGGAGG	
dmrt1-KO-S-R	CCTGTGCGGCTGTGTGTGGTC	
gsdf-KO-S-F	TTGTCGCTACTATCTCCTCCTC	
gsdf-KO-S-R	ATGTCTCTGTGCAGCTCTCC	
AMH-F5	ATGGCTCCGAGACCTTGACTG	Sexing
AMH-R3	CAGAAATGTAGACGCCCAGGTAT	

Supplementary Table S2. Masculinization rate of WT XX fish and different mutants by MT treatment and AI&MT treatment

Treatment type and fish genotype	Number of fish examined	Number of fish masculinized	masculinization rate (%)
MT-treated WT XX	43	43	100%
MT-treated XY *amhy*$^{-}$	28	28	100%
MT-treated XX/XY *gsdf*$^{-/-}$	13	13	100%
MT-treated XX/XY *dmrt1*$^{-/-}$	15	0	0%
AI&MT-treated WT XX	8	8	100%
AI&MT-treated XX/XY *dmrt1*$^{-/-}$	14	0	0%
